# DRP1 Inhibition Enhances Venetoclax-Induced Mitochondrial Apoptosis in TP53-Mutated Acute Myeloid Leukemia Cells through BAX/BAK Activation

**DOI:** 10.3390/cancers15030745

**Published:** 2023-01-25

**Authors:** Ji Eun Jang, Doh Yu Hwang, Ju-In Eom, June-Won Cheong, Hoi-Kyung Jeung, Hyunsoo Cho, Haerim Chung, Jin Seok Kim, Yoo Hong Min

**Affiliations:** 1 Division of Hematology, Department of Internal Medicine, Yonsei University College of Medicine, 50-1 Yonsei-ro, Seodaemun-gu, Seoul 03722, Republic of Korea; 2 Blood Cancer Research Institute, Yonsei University College of Medicine, 50-1 Yonsei-ro, Seodaemun-gu, Seoul 03722, Republic of Korea; 3 Division of Hemato-Oncology, Department of Internal Medicine, CHA Bundang Medical Center, CHA University, 59 Yatap-ro, Bundang-gu, Seongnam-si 13496, Gyeonggi-do, Republic of Korea

**Keywords:** TP53 mutation, DRP1 inhibition, venetoclax resistance, mitochondrial apoptosis, acute myeloid leukemia

## Abstract

**Simple Summary:**

Poor response to venetoclax in TP53-mutated (TP53mut) acute myeloid leukemia (AML) is a clinical challenge. To develop more effective therapeutic strategies for this subset of AML with dismal prognosis, the molecular mechanism involved in the development of venetoclax resistance needs to be elucidated. To the best of our knowledge, this study is the first to demonstrate that DRP1, a key player in the regulation of mitochondrial fission, is functionally involved in the development of venetoclax resistance in TP53mut AML. Inhibition of DRP1 with Mdivi-1 led to enhanced mitochondria-mediated cell death with the upregulation of BAX/BAK in TP53mut leukemia cell lines and primary leukemic blasts obtained from patients with TP53mut AML. Anti-apoptotic molecules, such as MCL-1 and BCL-xL, were reduced after combination treatment with venetoclax and Mdivi-1. These findings suggest that the combination of venetoclax with pharmacological DRP1 inhibition needs to be clinically evaluated for TP53mut AML.

**Abstract:**

Although TP53 mutations in acute myeloid leukemia (AML) are associated with poor response to venetoclax, the underlying resistance mechanism remains unclear. Herein, we investigated the functional role of dynamin-related protein 1 (DRP1) in venetoclax sensitivity in AML cells with respect to TP53 mutation status. Effects of DRP1 inhibition on venetoclax-induced cell death were compared in TP53-mutated (THP-1 and Kasumi-1) and TP53 wild-type leukemia cell lines (MOLM-13 and MV4-11), as well as in primary AML cells obtained from patients. Venetoclax induced apoptosis in TP53 wild-type AML cells but had limited effects in TP53-mutated AML cells. DRP1 expression was downregulated in MOLM-13 cells after venetoclax treatment but was unaffected in THP-1 cells. Cotreatment of THP-1 cells with venetoclax and a TP53 activator NSC59984 downregulated DRP1 expression and increased apoptosis. Combination treatment with the DRP1 inhibitor Mdivi-1 and venetoclax significantly increased mitochondria-mediated apoptosis in TP53-mutated AML cells. The combination of Mdivi-1 and venetoclax resulted in noticeable downregulation of MCL-1 and BCL-xL, accompanied by the upregulation of NOXA, PUMA, BAK, and BAX. These findings suggest that DRP1 is functionally associated with venetoclax sensitivity in TP53-mutated AML cells. Targeting DRP1 may represent an effective therapeutic strategy for overcoming venetoclax resistance in TP53-mutated AML.

## 1. Introduction

Venetoclax is a BH3 mimetic that facilitates apoptosis by selectively inhibiting the anti-apoptotic protein BCL-2 and freeing pro-apoptotic proteins that promote mitochondrial outer membrane permeabilization (MOMP) and cytochrome c release [1]. The addition of venetoclax to conventional hypomethylating therapy or low-dose cytarabine has advanced clinical outcomes for patients with acute myeloid leukemia (AML) who are elderly and unfit [2]; however, the development of resistance remains a challenge. The results of preclinical studies suggest that TP53 mutations confer resistance to venetoclax [3,4,5]. Critical mutations in the *TP53* gene are common in most cancers and are major contributors to cancer progression. The alteration of TP53 function is a negative prognostic factor for patients with AML [6,7]. TP53-mutated (TP53mut) AML is associated with poor outcomes and a short median overall survival of 5–9 months [7]. Thijssen et al. reported that TP53 deficiency reduced the activation of BAX and BAK in response to venetoclax, despite unchanged protein levels, leading to resistance to apoptosis [8]. TP53 directly interacts with BCL-2 family proteins in the mitochondrial outer membrane during a genotoxic response [9,10]. However, the mechanism through which TP53 activity affects venetoclax resistance remains unclear. Elucidation of the mechanisms involved in venetoclax resistance in TP53mut AML remains a critical area of unmet needs. An understanding of the influence of TP53 alterations on the response and resistance to venetoclax could provide therapeutic perspectives for such patients.

Mitochondrial dynamics play a key role in the maintenance of mitochondrial function, resulting in the regulation of signals for cell survival or death [11]. Previous studies have suggested the involvement of TP53 in mitochondrial dynamics and function [12,13,14]. Cumulative evidence has revealed a close link between cancer and unbalanced mitochondrial dynamics [15]. TP53-induced mitochondrial elongation is critical for the induction of senescence in various cancer cells [16]. Mitochondrial dynamics are primarily controlled by fusion- and fission-regulating proteins [17]. Dynamin-related protein 1 (DRP1), encoded by the *DNM1L* gene, regulates mitochondrial fission and contributes to the regulation of many biological processes, including mitochondrial biogenesis, the cell cycle, cell proliferation, and apoptosis [18]. Numerous reports have demonstrated that oncogenic signaling pathways are required for DRP1-mediated mitochondrial fission [19]. In addition, several studies have reported that the activation of DRP1-mediated fission is required for tumor migration and metastasis in breast, thyroid, brain, and prostate cancers [19,20,21,22]. However, the influence of TP53 on mitochondrial dynamics and the role of DRP1 in chemoresistance are yet to be investigated in AML.

Therefore, in the present study, we aimed to explore the functional roles of DRP1 in venetoclax sensitivity in TP53-wild-type and -mutated AML cells.

## 2. Materials and Methods

### 2.1. Cell Lines and Patient Samples

MOLM-13, MV4-11, THP-1, and Kasumi-1 human leukemia cell lines were obtained from the American Type Culture Collection (Manassas, VA, USA). The cells were cultured in Roswell Park Memorial Institute-1640 medium (Gibco, Thermo Fisher Scientific, Waltham, MA, USA) supplemented with 10% fetal bovine serum, 100 U/mL penicillin, and 100 μg/mL streptomycin (Gibco) at 37 °C in a humidified environment with 5% CO_2_. Primary samples were obtained from bone marrow (BM) aspirates taken from patients with TP53mut AML at diagnosis (*n* = 5) and from healthy donors who donated their BM aspirates for allogeneic hematopoietic stem cell transplantation (*n* = 5). The mutational status of the patients was assessed using next-generation sequencing, and sequencing libraries were prepared using genomic DNA extracted from BM aspirates. Sequencing libraries were hybridized with custom probes targeting 497 genes related to hematologic neoplasms (Table S1) and sequenced on a NextSeq 550Dx instrument (Illumina, San Diego, CA, USA). Samples from five patients with TP53 mutations were selected for further experiments. Demographics of the patients and healthy donors are described in Table S2. BM mononuclear cells (BMMCs) were isolated using Ficoll–Hypaque (GE Healthcare, Chicago, IL, USA) density gradient centrifugation as per the manufacturer’s instructions.

This study was conducted in accordance with the principles of the Declaration of Helsinki (2013) and approved by the Institutional Review Board of Severance Hospital (Yonsei University College of Medicine, Seoul, Republic of Korea; 4-2010-0732). The patient cohort was registered at ClinicalTrials.gov (NCT02344966), and all patients and healthy donors provided written informed consent.

### 2.2. Cell Culture and Treatment

Stock solutions of venetoclax, mitochondrial division inhibitor-1 (Mdivi-1), pifithrin-α, and NSC59984 (Selleckchem, Houston, TX, USA) were prepared in dimethyl sulfoxide (DMSO), and serial dilutions were prepared in the culture medium prior to each experiment. The final DMSO concentration was less than 0.2% (*v*/*v*) in all experiments. Cells in the logarithmic growth phase (1 × 10^5^ cells/mL) were exposed to different concentrations of chemicals.

### 2.3. Reagents and Antibodies

Rabbit polyclonal antibodies against caspase-3, poly (ADP-ribose) polymerase (PARP), MCL-1, phospho-MCL-1 (T163), BIM, BAK, BAX, phospho-AKT (S437), DRP1, phospho-DRP1 (S616), OPA1, and TP53, and horseradish peroxidase-conjugated goat anti-rabbit IgG and goat anti-mouse IgG were purchased from Cell Signaling Technology (Danvers, MA, USA). Rabbit polyclonal antibodies against BCL-xL and mouse monoclonal antibodies against BCL-2, FIS1, BAX, NOXA, and β-actin were obtained from Santa Cruz Biotechnology (Dallas, TX, USA). Rabbit polyclonal antibodies against phospho-DRP1 (S637), MFF, and MUL1, and a mouse monoclonal antibody against Cox IV were obtained from Abcam (Cambridge, UK). Mouse anti-cytochrome c monoclonal antibody was obtained from BD Pharmingen (San Diego, CA, USA). Details regarding each antibody are presented in Table S3.

### 2.4. Apoptosis Determination

An annexin-V assay was performed as described previously [23] using an LSR Fortessa flow cytometer (BD Biosciences, Franklin Lakes, NJ, USA). Cells were treated with various concentrations of venetoclax or other drugs for 48 h, resuspended in annexin-V binding buffer, and incubated with annexin-V/FITC (BD Biosciences) and propidium iodide (PI; Beckman Coulter, Brea, CA, USA) for 15 min before flow cytometry analysis. Data were analyzed using the FACSuite software v1.0.6. (BD Biosciences).

### 2.5. Analysis of Mitochondrial Membrane Potential

Mitochondrial membrane potential (MMP) in cells was measured using the MitoProbe^TM^ TMRM kit (Invitrogen, Waltham, MA, USA). For each condition, cells were resuspended in cell culture medium at approximately 1 × 10^6^ cells/mL, and 1 μL of the 20 μM stock TMRM reagent solution (20 nM final concentration) was added and incubated for 30 min at 37 °C and 5% CO_2_. The cells were washed once in 1 mL of Dulbecco’s phosphate-buffered saline (DPBS), resuspended in 500 μL of DPBS, and analyzed using a LSR Fortessa flow cytometer (561 nm laser) and FACSuite software (BD Biosciences).

### 2.6. Preparation of Mitochondrial and Cytosolic Fractions

A cell fractionation assay was performed using a Cytosolic/Mitochondrial Fractionation Kit (BioVision, Milpitas, CA, USA). Briefly, cells were harvested and washed with ice-cold DPBS, and the cell pellets were incubated on ice in 100 μL of 1X cytosol extraction buffer mix containing DTT and protease inhibitors for 10 min. After homogenization, the cells, large plasma membranes, and nuclei were removed using centrifugation at 700× *g* for 10 min at 4 °C. The supernatant was then centrifuged at 10,000× *g* for 30 min at 4 °C, and the resulting supernatant was collected as the cytosolic fraction. The pellet fraction containing the mitochondria was dissolved in 100 μL mitochondria extraction buffer mix containing DTT and protease inhibitors, vortexed for 10 s, and collected as the mitochondrial fraction.

### 2.7. Western Blot Analysis

Total cell lysates were prepared and analyzed using Western blotting as described previously [23]. Protein samples were recovered in sodium dodecyl sulfate (SDS) buffer and separated via SDS-polyacrylamide gel electrophoresis. Twenty micrograms of protein was loaded per lane in the Western blots. The separated proteins were transferred to nitrocellulose membranes and incubated with the appropriate primary and secondary antibodies. Protein bands were detected using enhanced chemiluminescence (GE Healthcare). β-Actin was used as the loading control.

### 2.8. Small-Interfering RNA (siRNA) Transfection

*DNM1L/Drp1*-specific siRNAs were purchased from Santa Cruz Biotechnology. Leukemia cells (2 × 10^6^) were directly transfected with siRNA (100 nM) using program V-01 of the Amaxa Nucleofector 2b device (Lonza Cologne GmbH, Colonge, Germany), according to the manufacturer’s instructions. After electroporation, the cells were resuspended in complete medium and incubated at 37 °C in a humidified atmosphere containing 5% CO_2_. Control cells were transfected with scrambled siRNA. The cells were then treated with the indicated concentrations of venetoclax and collected for Western blotting and apoptosis analyses.

### 2.9. Assessment of Intracellular Reactive Oxygen Species (ROS) Production

Intracellular ROS generation was measured as previously described [24]. Briefly, cells were treated with a given drug alone or in combination with the antioxidant *N*-acetylcysteine ((R)-2-acetamido-3-sulfanylpropanoic acid; Sigma-Aldrich, St. Louis, MO, USA) after pre-incubation with 10 μM dichlorodihydrofluorescein diacetate (DCFH-DA; Invitrogen, Carlsbad, CA, USA). In addition, 1 × 10^5^ cells were stained with DCFH-DA, washed, and resuspended in DPBS (Gibco). The amount of dihydrofluorescein was measured using flow cytometry.

### 2.10. Statistical Analyses

All values are presented as mean ± standard deviation of at least three independent experiments. Means of two groups were compared using a two-tailed Student’s *t*-test with the Prism software (GraphPad Prism 5.03, San Diego, CA, USA). Statistical significance was set at *p* < 0.05.

## 3. Results

### 3.1. Venetoclax Induces Apoptosis in Leukemia Cells According to TP53 Mutation Status

**Figure 1:**
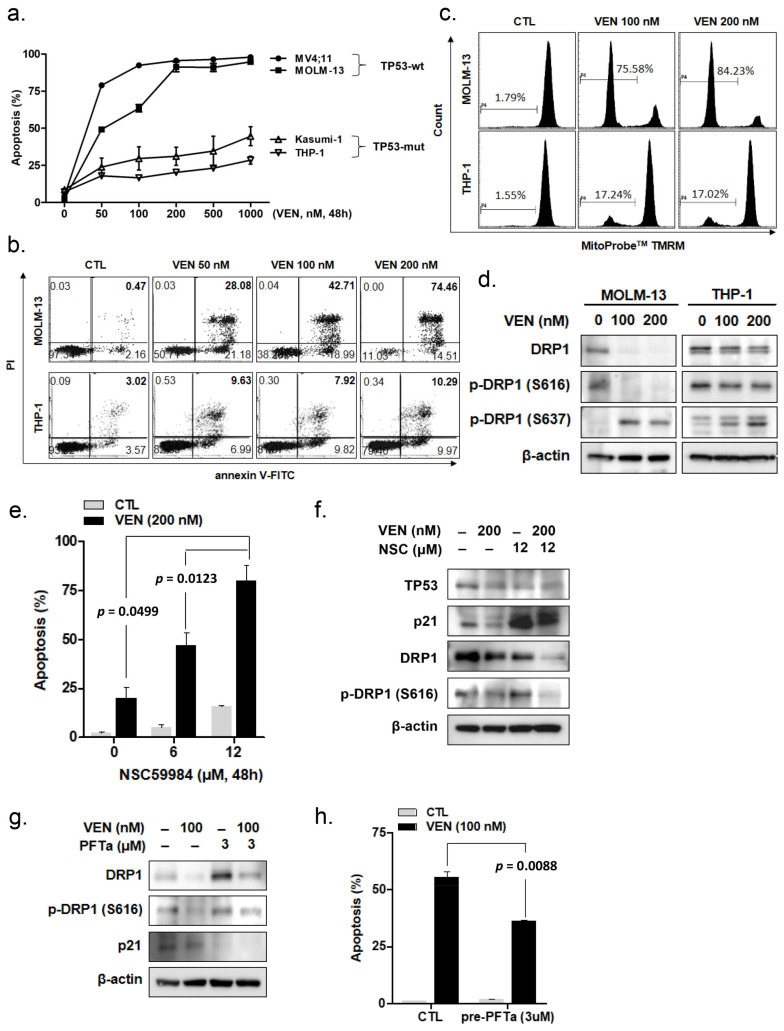
Effects of venetoclax in TP53-wild-type (TP53wt) and TP53-mutant (TP53mut) leukemia cells. (**a**,**b**) TP53wt leukemia cell lines (MV4-11 and MOLM-13) and TP53mut leukemia cell lines (Kasumi-1 and THP-1) were treated with various concentrations of venetoclax for 48 h. (**a**) The fraction of apoptotic cells in AML cell lines treated with venetoclax was analyzed using flow cytometry based on annexin-V/PI exclusion. Data are expressed as the mean ± standard deviation (SD) of three independent experiments. (**b**) Representative scatter-plot analysis of MOLM-13 and THP-1 cells. (**c**) Loss of mitochondrial membrane potential following TMRM staining in MOLM-13 and THP-1 cells treated with various concentrations of venetoclax for 48 h, as detected using flow cytometry. (**d**) MOLM-13 and THP-1 cells were treated with various concentrations of venetoclax for 48 h. Cell lysates were subjected to Western blotting using the indicated antibodies. β-Actin was used as a loading control. (**e**) THP-1 cells were treated with various concentrations of NSC59984 for 48 h in the presence or absence of 200 nM venetoclax. The apoptotic fraction was measured using flow cytometry based on annexin-V/PI exclusion. Data are the mean ± SD of three independent experiments. (**f**) THP-1 cells were treated with 200 nM venetoclax for 48 h in the presence or absence of 12 μM NSC59984. Cell lysates were subjected to Western blotting using the indicated antibodies. β-Actin was used as a loading control. (**g**,**h**) MOLM-13 cells were pretreated with pifithrin-α for 3 h and then treated with 200 nM venetoclax for 48 h. (**g**) Cell lysates were subjected to Western blotting using the indicated antibodies. β-Actin was used as a loading control. (**h**) The apoptotic fraction was measured using flow cytometry based on annexin-V/PI exclusion. Data are the mean ± SD of three independent experiments. CTL, control; Ven, venetoclax; PFTa, pifithrin-α.

First, we compared the effects of venetoclax on the induction of apoptosis in TP53-wild-type (TP53wt; MOLM-13 and MV4-11) and TP53mut (THP-1 and Kasumi-1) leukemia cell lines. As shown in Figure 1a, venetoclax treatment induced apoptosis in TP53wt leukemia cells in a concentration-dependent manner. By contrast, the extent of venetoclax-induced apoptosis was markedly lower in the TP53mut leukemia cell lines. Representative dot plot analyses showed that the frequency of annexin-V-positive cells was notably increased in a concentration-dependent manner in the TP53wt MOLM-13 cells (Figure 1b). However, the fraction of annexin-V-positive cells was approximately 10% in the TP53mut THP-1 cells and did not increase with a higher concentration of venetoclax (Figure 1b), suggesting that TP53 mutation is one of the molecular abnormalities associated with venetoclax resistance in AML cells. Additionally, we measured MMP disruption in MOLM-13 and THP-1 cells 48 h after venetoclax treatment. MMP disruption was markedly lower in the THP-1 cells than in the MOLM-13 cells (Figure 1c), suggesting that venetoclax resistance in TP53mut AML cells is related to lower mitochondria-mediated cell death.

### 3.2. Venetoclax Induces DRP1 Alteration According to TP53 Mutation Status

We investigated changes in DRP1 protein expression following venetoclax treatment. In MOLM-13 cells, venetoclax reduced the protein levels of DRP1 and phosphorylated DRP1 (phosphorylated at S616; p-DRP1 S616; Figure 1d). By contrast, changes in the levels of DRP1 and p-DRP1 S616 were negligible in THP-1 cells. Venetoclax treatment increased the phosphorylation of the DRP1 protein at S637 (p-DRP1 S637) in both MOLM-13 and THP-1 cells. These findings suggest that venetoclax-mediated downregulation of DRP1 and p-DRP1 S616 is associated with both venetoclax sensitivity and TP53 mutational status. We examined the effects of TP53 function on venetoclax-induced cell death and changes in DRP1 expression in TP53mut leukemia cells. When cells were treated with a combination of NSC59984, which restores the TP53 signaling pathway specifically in TP53mut cells [25], and 200 nM of venetoclax, the apoptotic cell fraction increased significantly in THP-1 cells (Figure 1e). Upregulation of endogenous protein levels of p21, a target molecule of TP53, with NSC59984 treatment in the presence or absence of venetoclax (Figure 1f and Figure S1) indicated increased activity of the TP53 signaling pathway in THP-1 cells, which have a constitutive loss of function of TP53. When cells were treated with NSC59984 and venetoclax, the levels of DRP1 and p-DRP1 S616 were substantially reduced (Figure 1f). Cleaved caspase-3 and PARP were detected after the combined treatment of venetoclax and NSC59984 but not with venetoclax alone (Figure S2). These findings suggest that TP53 pathway activation is associated with enhanced venetoclax-induced apoptosis with the downregulation of DRP1 expression, and mutated TP53 allows DRP1 to remain upregulated in the TP53mut AML cells. Thus, DRP1 downregulation is suggested to be requisite for venetoclax-mediated induction of apoptosis, and the functional status of TP53 is indispensable for the execution of this cellular process. To confirm the same, we examined the effects of pifithrin-α, a specific TP53 inhibitor, on venetoclax-induced apoptosis in MOLM-13 cells. Consistently, p21 protein levels were reduced after pretreatment with 3 μmol/L pifithrin-α, which inhibited venetoclax-induced downregulation of DRP1 expression in MOLM-13 cells (Figure 1g). In addition, pretreatment of MOLM-13 cells with pifithrin-α significantly suppressed venetoclax-induced apoptosis (*p* = 0.0088; Figure 1h).

### 3.3. DRP1 Inhibition Enhances Venetoclax-Induced Apoptosis in TP53mut Leukemia Cells

**Figure 2:**
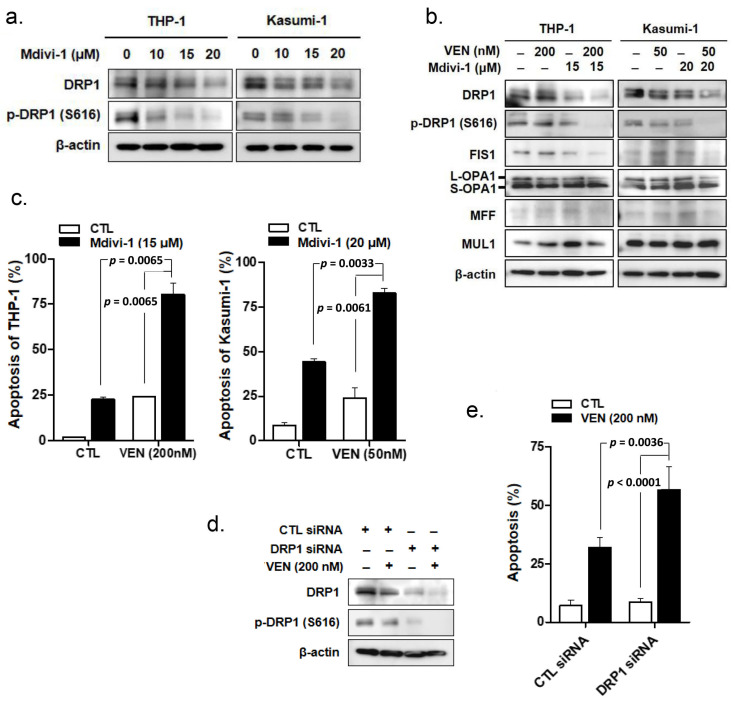
DRP1 inhibition enhances venetoclax-induced apoptosis in TP53mut leukemia cells. (**a**) THP-1 and Kasumi-1 cells were treated with various concentrations of Mdivi-1 for 24 h. Cell lysates were subjected to Western blotting using the indicated antibodies. β-Actin was used as a loading control. (**b**) THP-1 cells were treated with 200 nM venetoclax in the presence or absence of 15 μM Mdivi-1, and Kasumi-1 cells were treated with 50 nM venetoclax for 48 h in the presence or absence of 20 μM Mdivi-1. Cell lysates were subjected to Western blotting using the indicated antibodies. β-Actin was used as a loading control. (**c**) The apoptotic fraction was measured using flow cytometry based on annexin-V/PI exclusion in THP-1 cells ((**c**), left panel) treated with 200 nM venetoclax in the presence or absence of 15 μM Mdivi-1 and Kasumi-1 cells ((**c**), right panel) treated with 50 nM venetoclax for 48 h in the presence or absence of 20 μM Mdivi-1. Data are the mean ± SD of three independent experiments. (**d**) THP-1 cells were transfected with *DNM1L/Drp1* siRNA or control siRNA. After incubation of transfected cells with 200 nM venetoclax for 48 h, cell lysates were subjected to Western blotting using the indicated antibodies. β-Actin was used as a loading control. (**e**) THP-1 cells were transfected with *DNM1L/Drp1* siRNA or control siRNA. Transfected cells were incubated with 200 nM venetoclax for 48 h. The apoptotic fraction was measured using flow cytometry analysis. CTL, control; Ven, venetoclax.

Since TP53 mutation cannot effectively downregulate the expression of DRP1, which is crucial for venetoclax-induced apoptosis, it is tempting to pharmacologically inhibit DRP1 to bypass TP53 mutational effects and enhance the effects of venetoclax. Therefore, we investigated the effects of Mdivi-1, a selective inhibitor of DRP1, on the levels of DRP1 and p-DRP1 S616 in TP53mut AML cell lines. As shown in Figure 2a, Mdivi-1 decreased the levels of DRP1 and p-DRP1 S616 in both THP-1 and Kasumi-1 cells in a concentration-dependent manner. The combination of Mdivi-1 and venetoclax resulted in the further downregulation of DRP1 and p-DRP1 S616 levels in THP-1 and Kasumi-1 cells, compared to that seen with venetoclax treatment alone (Figure 2b). With venetoclax treatment, the expression of other molecules involved in the regulation of mitochondrial dynamics, such as FIS1, L/S-OPA1, MFF, and MUL1, showed no discernable changes in the presence or absence of Mdivi-1 (Figure 2b). When cells were treated with both Mdivi-1 and venetoclax (200 nM for THP-1 and 50 nM for Kasumi-1), the fractions of apoptotic cells were significantly increased in these cell lines (*p* = 0.0065 for THP-1 and *p* = 0.0061 for Kasumi; Figure 2c). Mdivi-1 concentrations of 15 and 20 μM were selected for experiments with THP-1 and Kasumi-1 cells, respectively (Figure S3). The combined effects of venetoclax and Mdivi-1 on the induction of apoptosis were synergistic, with combination index (CI) values less than 1 (Figure S4). The enhanced effect of DRP1 inhibition on venetoclax-induced apoptosis was reproduced in *DNM1L/Drp1* knockdown experiments using anti-*DNM1L/Drp1* siRNA in THP-1 cells (Figure 2d); the fraction of venetoclax-induced apoptotic cells was significantly higher in *DNM1L/Drp1*-silenced THP-1 cells than in control siRNA-transfected THP-1 cells (*p* = 0.0036, Figure 2e). These findings strongly suggest that DRP1 inhibition, either by the pharmacological or genetic approach, could effectively increase venetoclax-induced apoptosis in TP53mut AML cells.

### 3.4. DRP1 Inhibition Increases Venetoclax-Induced Apoptosis of Primary Leukemic Blasts Obtained from Patients with TP53mut AML

**Figure 3:**
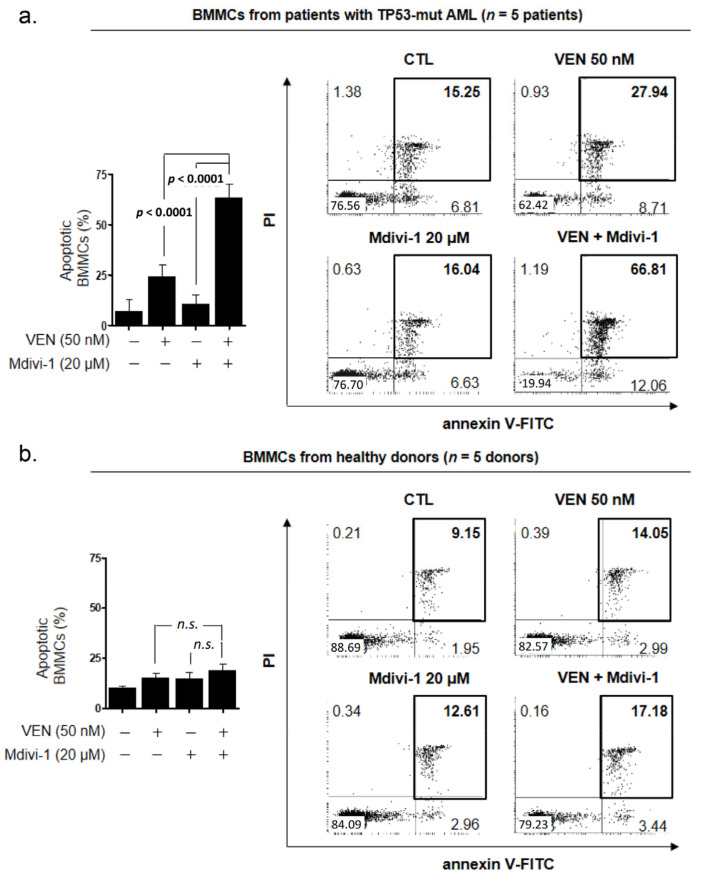
Sensitization of TP53mut AML cells, but not normal bone marrow mononuclear cells (BMMCs), to venetoclax-induced apoptosis via DRP1 inhibition. (**a**,**b**) BMMCs from patients with TP53mut AML (**a**, *n* = 5) and healthy donors (**b**, *n* = 5) were treated with 50 nM venetoclax in the presence or absence of 20 μM Mdivi-1 for 48 h. Subsequently, apoptosis levels (a and b, left panel) were measured using flow cytometry based on annexin-V/PI exclusion. Data are the mean ± SD. Representative scatter-plot analysis ((**a**,**b**), right panel) of BMMCs. AML, acute myeloid leukemia; CTL, control; Ven, venetoclax.

We also examined the effects of Mdivi-1 on venetoclax-induced apoptosis in primary leukemic blasts obtained from the BM of untreated patients with TP53mut AML. Primary AML blasts were treated with 50 nM of venetoclax, in the presence or absence of 20 μM Mdivi-1, for 48 h (Table S2). As shown in Figure 3a (left), Mdivi-1 significantly increased venetoclax-induced apoptosis (24.16 ± 2.71% for venetoclax alone and 63.47 ± 3.03% for venetoclax plus Mdivi-1; *p* < 0.0001). Representative dot plot analyses of one case showed that the fraction of annexin-V/PI-positive cells markedly increased with the combined treatment of Mdivi-1 and venetoclax (Figure 3a, right). Notably, the addition of Mdivi-1 did not affect venetoclax-induced apoptosis in normal BMMCs (Figure 3b), suggesting that this combination therapy can effectively eliminate leukemia cells without compromising normal hematopoiesis.

### 3.5. Effects of DRP1 Inhibition on the Mitochondrial Apoptosis Pathway in TP53mut AML

**Figure 4:**
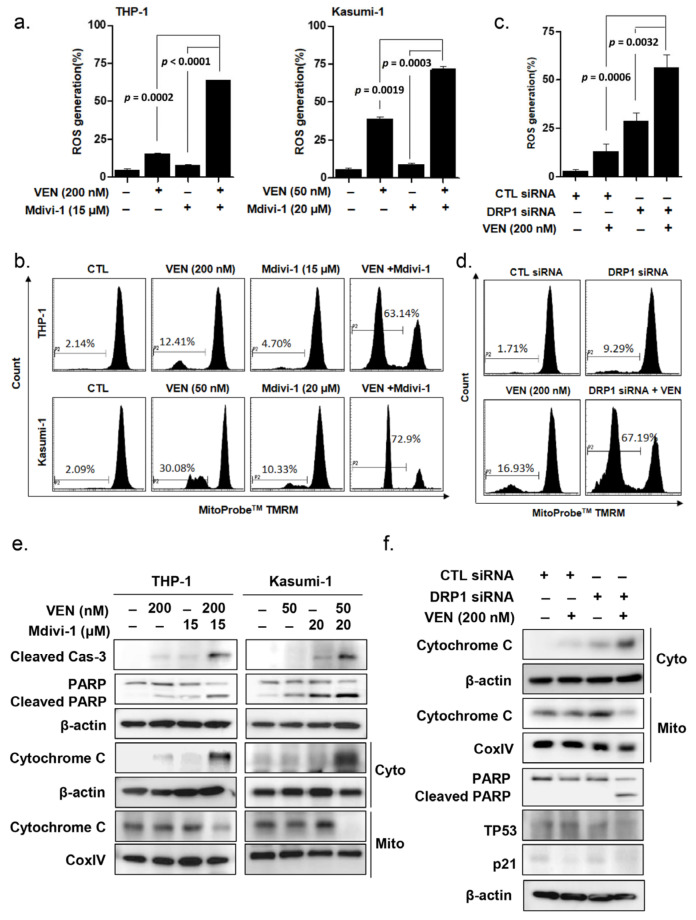
DRP1 inhibition enhances venetoclax-induced ROS generation and mitochondria-mediated apoptosis in TP53mut leukemia cells. (**a**–**c**) THP-1 and Kasumi-1 cells were treated with 200 or 50 nM venetoclax in the presence or absence of 15 or 20 μM Mdivi-1, respectively. (**a**) After incubation for 24 h, intracellular ROS generation was measured using flow cytometry analysis. Data are the mean ± SD of three independent experiments. (**b**) After incubation for 48 h, the mitochondrial membrane potential in THP-1 and Kasumi-1 cells loaded with TMRM was detected using flow cytometry. (**c**,**d**) THP-1 cells were transfected with DRP1 siRNA or CTL siRNA after incubation of transfected cells with 200 nM venetoclax. (**c**) After incubation for 24 h, intracellular ROS generation was measured using flow cytometry analysis. (**d**) After incubation for 48 h, mitochondrial membrane potential in THP-1 cells loaded with TMRM was detected using flow cytometry. (**e**) After incubation for 48 h, the cytosolic (Cyto) and mitochondrial (Mito) fractions were prepared and subjected to Western blotting using an antibody against cytochrome C. Total cell lysates were subjected to Western blotting using the indicated antibodies. β-Actin was used as a loading control. COX IV and β-Actin were used as mitochondrial and cytosolic marker proteins, respectively. (**f**) THP-1 cells were transfected with *DNM1L/Drp1* siRNA or control siRNA. After incubation of transfected cells with 200 nM venetoclax for 48 h, the Cyto and Mito fractions were prepared and subjected to Western blotting using an antibody against cytochrome C. Total cell lysates were subjected to Western blotting using PARP antibodies. β-Actin was used as a loading control. COX IV and β-Actin were used as mitochondrial and cytosolic marker proteins, respectively. CTL, control; ROS, reactive oxygen species; Ven, venetoclax.

The changes in the generation of intracellular ROS were evaluated after treatment of TP53mut AML cells with Mdivi-1 and venetoclax. As shown in Figure 4a, the addition of Mdivi-1 to venetoclax led to a significant increase in the extent of intracellular ROS generation compared to that observed with venetoclax alone in TP53mut AML cells (*p* = 0.0002 for THP-1 and *p* = 0.0019 for Kasumi-1 cells). Increased disruption of the MMP was also observed with the combined treatment of Mdivi-1 and venetoclax (Figure 4b). Consistently, *DNM1L/Drp1* knockdown also considerably increased intracellular ROS generation (Figure 4c) and MMP disruption in THP-1 cells (Figure 4d). Moreover, cleavage of caspase-3 and PARP became evident with the combined treatment of Mdivi-1 and venetoclax in THP-1 and Kasumi-1 cells (Figure 4e). Mdivi-1 treatment (Figure 4e) or *DNM1L/Drp1*-silencing (Figure 4f) considerably increased the extent of cytosolic cytochrome C release from the mitochondria. β-Actin and COX IV were used as loading controls for the cytosolic and mitochondrial fractions, respectively. These findings indicate that DRP1 inhibition enhances venetoclax-induced apoptosis through activation of the mitochondrial apoptotic pathway in TP53mut AML cells.

### 3.6. Effects of DRP1 Inhibition on the Regulatory Pathway of Mitochondrial Apoptosis

**Figure 5:**
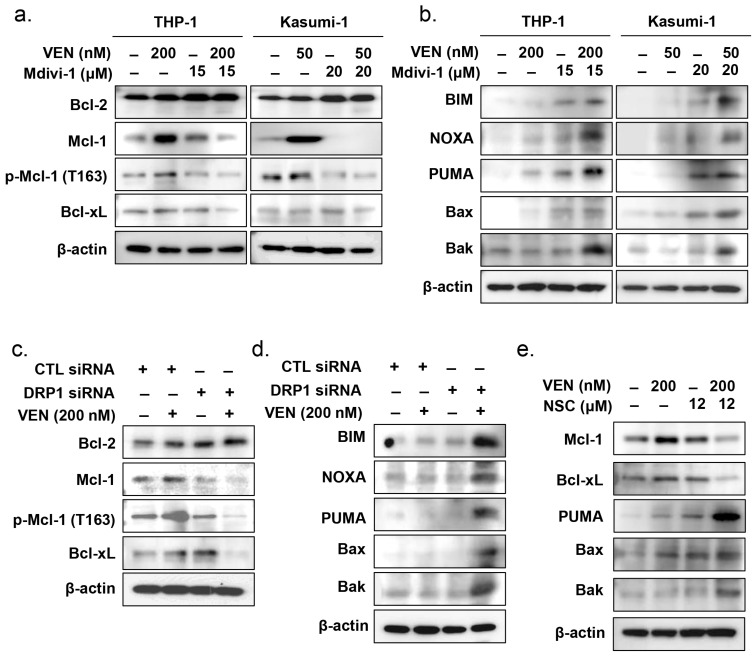
TP53-induced DRP1 inhibition reinforces BAX activation by downregulating MCL-1 and BCL-xL expression. (**a**,**b**) THP-1 and Kasumi-1 cells were treated with 200 or 50 nM venetoclax for 48 h in the presence or absence of 15 or 20 μM Mdivi-1, respectively; cell lysates were subjected to Western blotting using the indicated antibodies. β-Actin was used as a loading control. (**c**,**d**) THP-1 cells were transfected with *DNM1L/Drp1* siRNA or control siRNA. After incubation of transfected cells with 200 nM venetoclax for 48 h, cell lysates were subjected to Western blotting using the indicated antibodies. (**e**) THP-1 cells were treated with 200 nM venetoclax for 48 h in the presence or absence of 12 μM NSC59984. Cell lysates were subjected to Western blotting using the indicated antibodies. β-Actin was used as a loading control. CTL, control; Ven, venetoclax.

We then examined changes in the expression of several BCL-2 family proteins after treatment of TP53mut AML cells with venetoclax in the presence or absence of DRP1 inhibition. BCL-2 protein levels were not altered by venetoclax and/or Mdivi-1 treatment in TP53mut AML cells (Figure 5a). MCL-1 and to a lesser extent p-MCL-1 T163 levels were upregulated by venetoclax. However, the combination treatment of Mdivi-1 and venetoclax markedly decreased MCL-1 and p-MCL-1 T163 protein levels. Downregulation of BCL-xL was observed with the combined treatment of Mdivi-1 and venetoclax (Figure 5a and Figure S5a). Moreover, BIM, NOXA, PUMA, BAX, and BAK levels were markedly upregulated following combined treatment of venetoclax and Mdivi-1 in both THP-1 and Kasumi-1 cells (Figure 5b and Figure S5b). These findings were consistent with the results of *DNM1L/Drp1* knockdown experiments using anti-*DNM1L/Drp1* siRNA. When *DNM1L/Drp1*-silenced THP-1 cells were treated with venetoclax, the levels of MCL-1, p-MCL-1 (T163), and BCL-xL were markedly decreased (Figure 5c), whereas those of BIM, NOXA, PUMA, BAX, and BAK were notably upregulated (Figure 5d). MCL-1 and BCL-xL levels were notably downregulated with the addition of the TP53 activator NSC59984 along with venetoclax in THP-1 cells, whereas PUMA, BAX, and BAK were upregulated (Figure 5e). The findings observed with the combination of NSC59984 and venetoclax in TP53mut AML cells are consistent with the results of the combined venetoclax treatment and DRP1 inhibition. Since DRP1 inhibition could potentially restore the functional aspects of TP53, DRP1 inhibition in combination with venetoclax treatment could potentially improve the therapeutic efficacy of venetoclax in TP53mut AML. Moreover, considering that normal BM cells are protected from the synergistic effects of venetoclax and DRP1 inhibition, this combination could provide a promising therapeutic strategy for TP53mut AML.

## 4. Discussion

TP53 mutations have been associated with therapeutic resistance to venetoclax [3,4,5,26]. However, the mechanism by which TP53 activity regulates the apoptosis-inducing effects of venetoclax is unclear. To the best of our knowledge, this study is the first to demonstrate that DRP1, one of the key molecules that regulate mitochondrial dynamics, plays a critical role in modulating venetoclax-induced mitochondrial apoptosis in TP53mut AML cells. Loss-of-function TP53 mutations resulted in the dysregulation of DRP1 activity, which needs to be downregulated for venetoclax-mediated induction of apoptosis in AML.

Nuclear TP53 accumulation regulates genes associated with the mitochondrial apoptotic pathway, which is stimulated by cellular stress signals [27]. Activation of the intrinsic apoptotic pathway by TP53 is associated with upregulation of NOXA and PUMA, which are BCL-2 family proteins that interact with BAX and BAK in the mitochondria [28,29]. TP53 is also known to directly activate BAX/BAK [30]. Loss of TP53 has been identified as an important factor that is involved in the development of venetoclax resistance through a genome-wide CRISPR screen in AML [26,31]. Loss of TP53 leads to decreased expression and release of NOXA and PUMA proteins from BCL-2 or MCL-1, leading to reduced BAX/BAK activation in response to BH3-mimetic drugs [8]. These findings suggest that intact TP53 function is essential for sustaining responses to venetoclax in AML cells via BAX/BAK activation. However, the mechanism by which TP53 mutation regulates the apoptosis-inducing activity of venetoclax needs to be clarified further.

Mitochondrial dynamics are related to mitochondrial stress adaptation. DRP1, a cytosolic GTPase that is recruited to the mitochondrial surface in response to metabolic changes, stress, and growth factor stimulation, is the key protein that induces mitochondrial fission [18]. Accumulating evidence shows the pivotal role of mitochondrial fission in chemoresistance [32,33]. One of the signals involved in the regulation of DRP1 is TP53. TP53 promotes mitochondrial elongation through suppression of DRP1 [34]. Depletion of TP53 exaggerates DRP1-mediated mitochondrial fission [35]. In the present study, venetoclax-induced apoptosis was found to be associated with alterations in the levels of DRP1. Marked downregulation of DRP1 was demonstrated in venetoclax-sensitive TP53wt AML cells, whereas this was not observed in venetoclax-resistant TP53mut AML cells. To demonstrate that TP53 functional activity modulates venetoclax-induced cell death through the regulation of DRP1 levels, we used a combination treatment of venetoclax and a TP53 activator NSC59984 in TP53mut AML cells. The addition of NSC59984 restored TP53 function, which was evident from increased p21 levels, which subsequently resulted in a significant increase in venetoclax-mediated apoptosis as well as downregulation of DRP1 in TP53mut AML cells.

The mechanism through which DRP1 regulates the sensitivity of leukemia cells to venetoclax-induced cell death is unclear. Mitochondria play a critical role in apoptosis by releasing pro-apoptotic factors from the intermembrane space. This process, known as MOMP, is regulated tightly by BCL-2 family proteins. Pro-apoptotic BCL-2 family members, BAX and BAK, undergo a conformational change upon activation by BH3 domain-only proteins and MOMP, whereas anti-apoptotic BCL-2 members inhibit permeabilization [36,37]. Venetoclax induces structural alterations in the mitochondria, leading to disruptions in the outer mitochondrial membrane and activation of the intrinsic mitochondrial apoptotic pathway [38]. Loss of myeloblast sensitivity to venetoclax was reported to be accompanied by the loss of direct mitochondrial sensitivity to venetoclax-induced MOMP [39]. Although mitochondrial dynamics were not shown to directly affect MOMP, DRP1-mediated mitochondrial fission is thought to play a role in mitochondrial sensitivity to MOMP, since a close association between MOMP and mitochondrial dynamics for swift protein efflux from the mitochondria has been suggested [40].

There is an ongoing debate in the scientific community on the roles of mitochondrial dynamics and DRP1 in the regulation of mitochondrial apoptosis. DRP1-mediated fission may act during an early step in apoptosis, as DRP1 depletion delays cytochrome c release and subsequent apoptosis [41]. Mitochondrial DRP1 promotes BAX oligomerization, which leads to cytochrome c release from the mitochondria [42]. However, accumulating evidence suggests that DRP1-mediated mitochondrial fission plays an anti-apoptotic role [43]. Cells with hyperfragmented mitochondria were defective in BAX-induced MOMP, which was rescued by DRP1 inhibition [44]. Notably, small mitochondria resist MOMP [45]. Tumor cells often have hyperfragmented mitochondria; in these cells, elevated DRP1 activity was associated with the extent of proliferation and stemness [46]. DRP1 inhibition was shown to enhance apoptosis in several tumor cells [32,47,48]. In acute leukemia cells, DRP1-dependent mitochondrial fragmentation downregulates mitochondrial ROS levels and promotes the glycolytic phenotypic switch, resulting in the development of chemoresistance [49]. This contradiction may be due to differences in cellular contexts and stimuli. Notably, in the present study, we found that DRP1 inhibition synergistically enhances venetoclax-induced mitochondrial apoptosis via activation of BAX/BAK in TP53mut AML cell lines as well as in primary AML cells; however, it does not affect normal BMMCs. The results of these studies indicate that DRP1 inhibition may provide an effective therapeutic approach for overcoming drug resistance in specific subsets of tumors, specifically in TP53mut AML. We further examined changes in the levels of key BCL-2 family members with DRP1 inhibition in TP53mut AML cells. The combination of a DRP1 inhibitor and venetoclax led to a marked increase in the levels of BAX, BAK, BIM, NOXA, and PUMA. By contrast, the levels of MCL-1 and BCL-xL, which are representative anti-apoptotic molecules involved in venetoclax resistance, were notably decreased. However, *DNM1L/Drp1* knockout alone did not induce these changes. It is worth noting that the correction of hyperfragmented mitochondria by DRP1 inhibition has been reported to lead to a balanced mitochondrial network, which further leads to apoptotic resensitization with BAX activation [44]. Recently, Chen et al. demonstrated that mitochondrial structure remodeling is associated with venetoclax resistance in AML [31]. Thus, we hypothesize that in response to cytotoxic stress that is induced by venetoclax treatment, TP53 inhibits DRP1 and alleviates DRP1-mediated mitochondrial hyperfragmentation, resulting in a mitochondrial structure that facilitates venetoclax-mediated activation of BAX/BAK, facilitating induction of MOMP. Further investigation is needed to understand the molecular mechanisms underlying the involvement of DRP1 inhibition in venetoclax-mediated BAX/BAK activation.

## 5. Conclusions

Although further investigation of the regulatory mechanism and molecular targets of TP53 involved in DRP1 regulation is needed to improve our knowledge of the mechanism underlying venetoclax resistance in TP53mut leukemia cells, this study is the first to report that TP53-mediated DRP1 inhibition plays a critical role in venetoclax-induced mitochondrial apoptosis. Our results indicate that targeting DRP1 in TP53mut leukemia cells may represent a novel strategy to overcome chemoresistance. Hence, combination therapy with DRP1 inhibitors and venetoclax may be a useful approach for the treatment of patients with TP53mut AML. Our findings may provide valuable insights into the molecular mechanism underlying venetoclax resistance in TP53mut leukemia cells, as well as present potential therapeutic targets.

## Data Availability

The analyzed data sets generated during the study are available from the corresponding author on reasonable request.

## Supplementary Materials

The following supporting information can be downloaded at: https://www.mdpi.com/article/10.3390/cancers15030745/s1, Table S1: List of 497 genes related to hematologic neoplasms; Table S2: Effects of Mdivi-1 on venetoclax-induced apoptosis in primary acute myeloid leukemia FLT3 cells and normal BMMCs; Table S3: Brand, catalog no., and dilution of each antibody; Figure S1: Western blot analysis of total cell extracts shows NSC59984 concentration-dependent (6, 12, 25, 50, and 100 μM for 8 h) increase in p21 levels in THP-1 cells; Figure S2: Cleaved caspase-3 and PARP after treatment of TP53mut AML cells with venetoclax in the presence or absence of NSC59984 in TP53mut AML cells; Figure S3: DRP1 inhibition enhances venetoclax-induced apoptosis by mitochondria in TP53-mut leukemia cells; Figure S4: ComfuSyn analysis to determine the synergistic effect of venetoclax and Mdivi-1 on THP-1 (a) and Kasumi-1 (b) cells; Figure S5: Expression of anti-apoptotic molecules and BCL-2 family proteins after treatment of TP53mut AML cells with venetoclax in the presence or absence of DRP1 inhibition.
