# Supplementary material for: DRP1 Inhibition Enhances Venetoclax-Induced Mitochondrial Apoptosis in TP53-Mutated Acute Myeloid Leukemia Cells through BAX/BAK Activation
Source: Cancers (Basel). 2023 Jan 25;15(3):745. doi: 10.3390/cancers15030745 (PMC9913445; doi:10.3390/cancers15030745)
Supplement: Supplementary file 1 [file cancers-15-00745-s001.zip › cancers-2174698-supplementary/Supplementary figures.pdf]

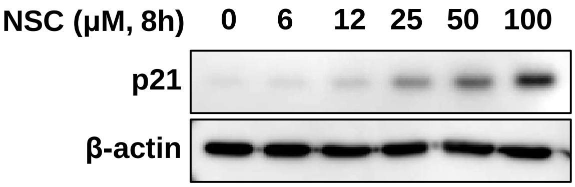

Figure S1. Western blot analysis of total cell extracts shows NSC59984 concentration-dependent (6, 12, 25, 50, and 100 μM for 8 h) increase in p21 levels in THP-1 cells. THP-1 cells were treated with various concentrations of NSC59984 for 8 h. Cell lysates were subjected to western blotting using the p21 antibodies.

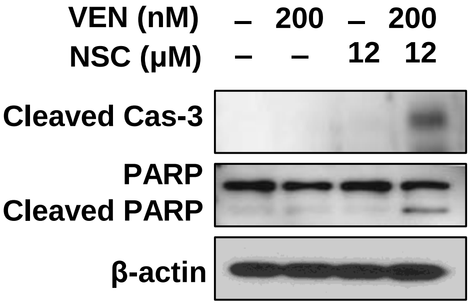

Figure S2. Western blot analysis of total cell extracts shows cleaved caspase-3 and PARP after treatment of TP53mut AML cells with venetoclax in the presence or absence of NSC59984 in TP53mut AML cells.

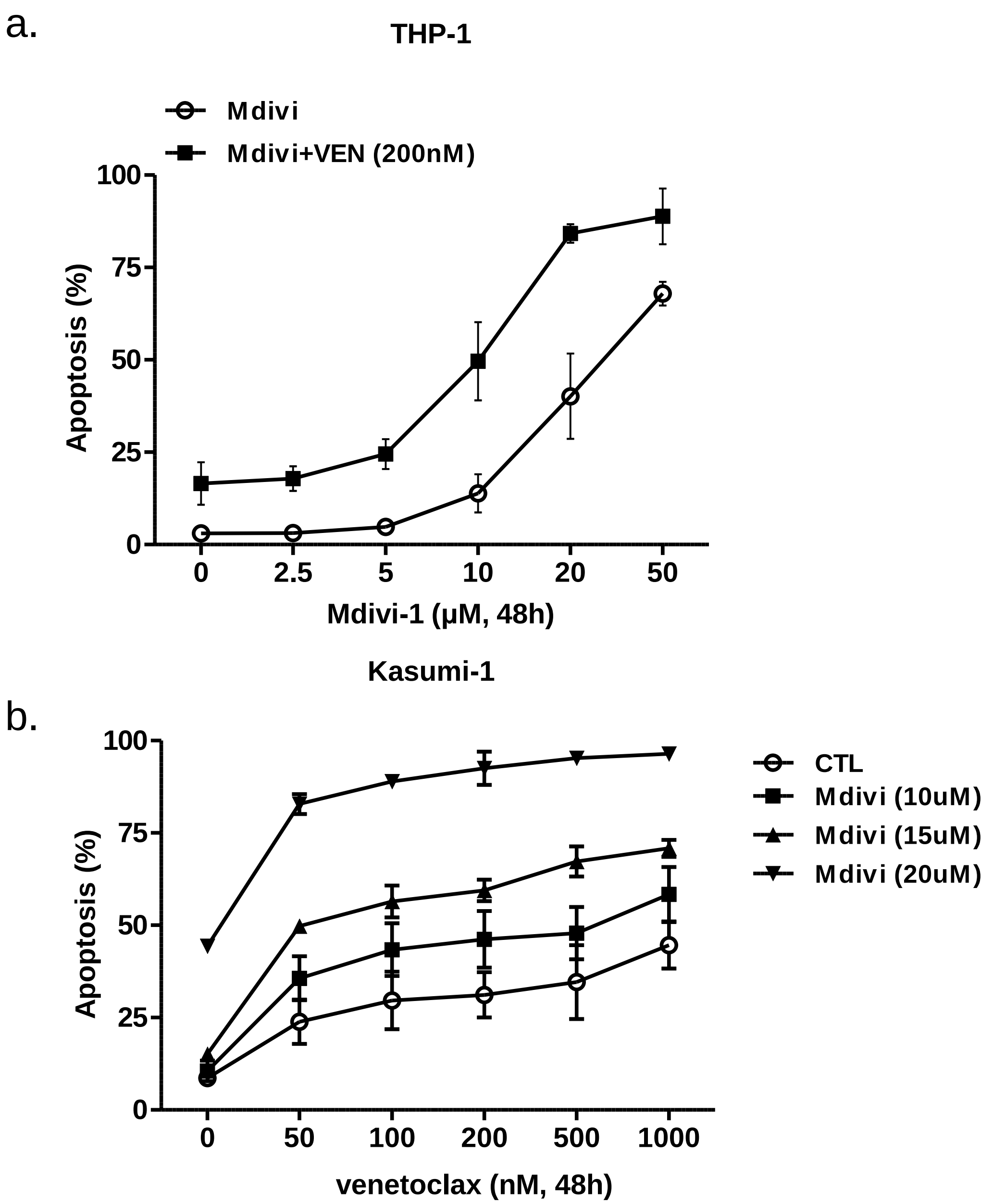

Figure S3. DRP1 inhibition enhances venetoclax-induced apoptosis by mitochondrial in TP53-mut leukemia cells. (a) The apoptotic fraction was measured by flow cytometry based on annexin-V/PI exclusion in THP-1 cells treated with 200 nM venetoclax in the presence or absence of various dose of Mdivi-1 for 48 h. (b) Kasumi-1 cells treated with 10 / 15 / 20 μM Mdivi-1 in the presence or absence of various dose of venetoclax for 48 h. Data are the means ± SDs from three independent experiments.

a.

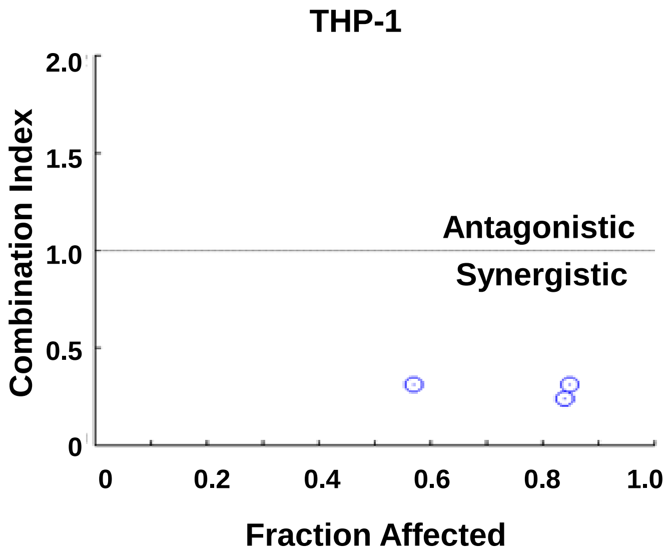

b.

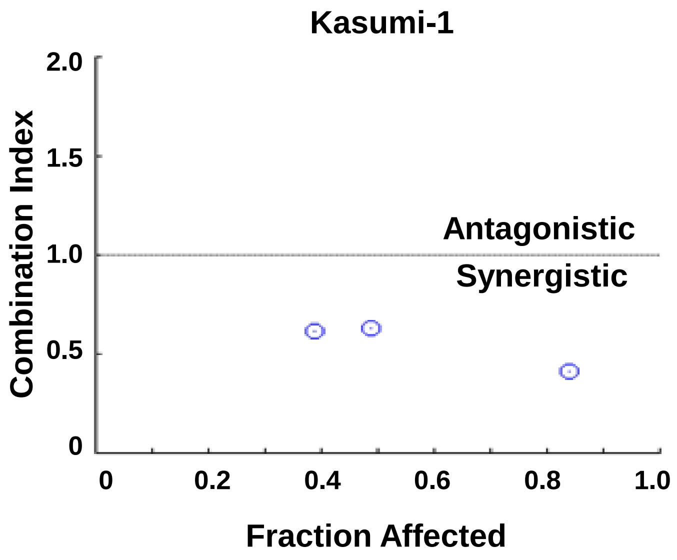

Figure S4. ComfuSyn analysis to determine the synergistic effect of venetoclax and Mdivi-1 on THP-1 (a) and Kasumi-1 (b) cells.

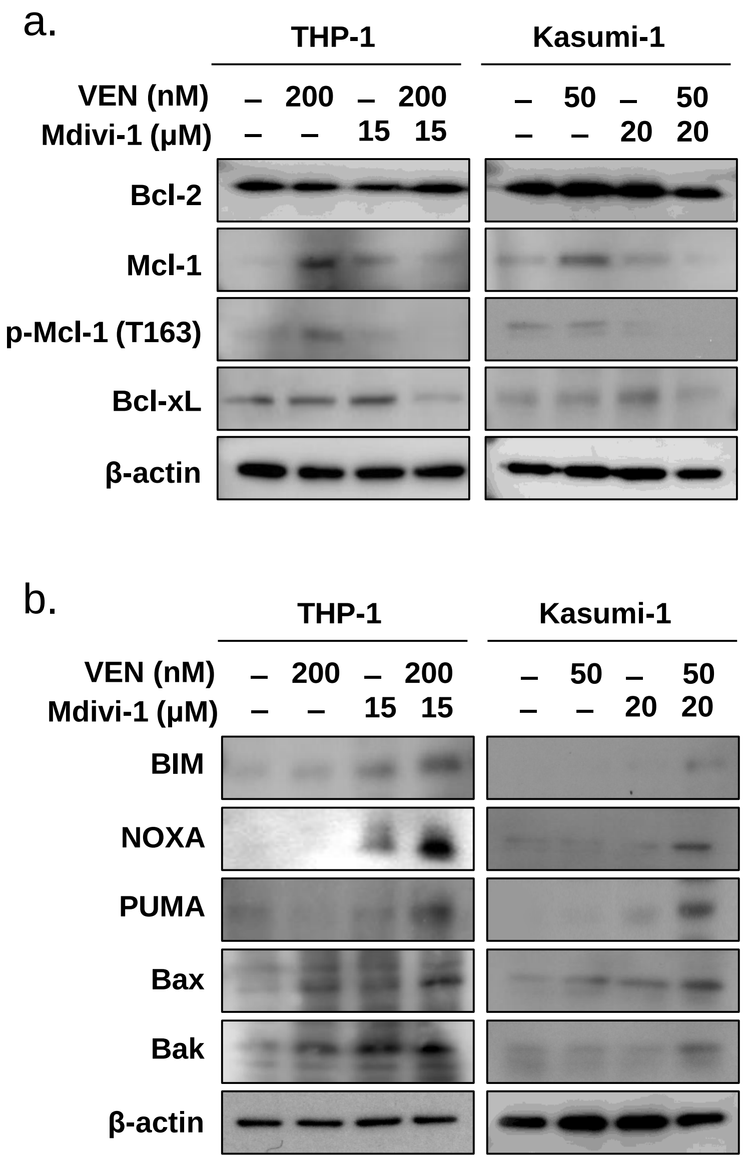

Figure S5. Western blot analysis of total cell extracts shows expressions of anti-apoptotic molecules and BCL-2 family proteins after treatment of TP53mut AML cells with venetoclax in the presence or absence of DRP1 inhibition.
