# Supplementary material for: DRP1 Inhibition Enhances Venetoclax-Induced Mitochondrial Apoptosis in TP53-Mutated Acute Myeloid Leukemia Cells through BAX/BAK Activation
Source: Cancers (Basel). 2023 Jan 25;15(3):745. doi: 10.3390/cancers15030745 (PMC9913445; doi:10.3390/cancers15030745)
Supplement: Supplementary file 1 [file cancers-15-00745-s001.zip › cancers-2174698-supplementary/Supplementary Tables.docx.pdf]

Table S1. List of 497 genes related to hematologic neoplasms

ABCB1, ABCB7, ABCG2, ABCG5, ABCG8, ABL1, ABL2, ACD, ACTB, ACTN1, ADA, ADAMTS13, AIRE, AK1, AK2, AKT2, ALAS2, ALDOA, AMN, ANK1, ANKRD26, AP3B1, ARID1A, ARPC1B, ASXL1, ATG2B, ATM, ATR, ATRX, AXIN1, BCL11B, BCL2, BCL6, BCOR, BCORL1, BHLHE41, BIRC3, BLM, BPGM, BRAF, BRCA1, BRCA2, BRCC3, BRINP3, BRIP1, BTG1, BTK, BTLA, C3, C4BPA, C4BPB, CALN1, CALR, CARD11, CASP10, CBL, CBLB, CBLC, CCND1, CD200, CD247, CD27, CD36, CD3D, CD3E, CD40LG, CD46, CD58, CD59, CD79B, CDAN1, CDKN1B, CDKN2A, CDKN2B, CEBPA, CFB, CFH, CFHR1, CFHR3, CFHR4, CFHR5, CFI, CHD1, CHD4, CHD9, CHMP2B, CLPB, CNOT3, COX4I2, CREBBP, CRLF2, CSF1R, CSF2RA, CSF3R, CTC1, CTCF, CTSC, CUBN, CUX1, CXCR4, CYB5R3, CYBA, CYBB, CYCS, DCLRE1C, DDX41, DGKE, DGKH, DHFR, DIS3, DKC1, DNM2, DNMT1, DNMT3A, EBF1, ECT2L, EED, EGFR, EGLN1, EGLN2, EGLN3, EHMT1, ELANE, EP300, EPAS1, EPB41, EPB42, EPCAM, EPO, EPOR, ERCC4, ERG, ETNK1, ETV6, EZH2, F2R, FANCA, FANCB, FANCC, FANCD2, FANCE, FANCF, FANCG, FANCI, FANCL, FANCM, FAS, FASLG, FAT1, FBXW7, FCGR1A, FCGR3B, FERMT3, FLI1, FLNA, FLT3, FOXP3, G6PC3, G6PD, GATA1, GATA2, GATA3, GCLC, GFI1, GFI1B, GIF, GINS1, GLRX5, GNAS, GNB1, GP1BA, GP1BB, GP9, GPI, GPRC5A, GPX1, GSKIP, GSN, GSR, GSS, HAX1, HBA1, HBA2, HBB, HBD, HCLS1, HFE, HIF1A, HIF1AN, HIF3A, HK1, HNRNPK, HOOK1, HOXA10, HOXA11, HRAS, HSPA9, HUWE1, ID3, IDH1, IDH2, IFNG, IFNGR1, IFNGR2, IKZF1, IKZF2, IKZF3, IL12RB1, IL2RB, IL2RG, IL3RA, IL7R, IRF1, ITGA2, ITGA2B, ITGB2, ITGB3, ITK, ITPKB, JAGN1, JAK1, JAK2, JAK3, JAKMIP2, JMJD1C, KDM5C, KDM6A, KDM7A, KIF23, KIT, KLF1, KMT2A, KMT2C, KMT2D, KRAS, LAMB4, LAMTOR2, LAPTM5, LCK, LEF1, LIG4, LMNA, LMO1, LMO2, LPIN2, LRP1B, LRRC4, LUC7L2, LYL1, LYST, MAD2L2, MAGT1,

MAP2K1, MAP2K2, MASTL, MBL2, MECOM, MED13, MEF2B, MEF2C, MEFV, MET, MLH1, MLLT10, MLLT3, MPL, MSH2, MSH4, MSH6, MTA1, MTAP, MTR, MTRR, MVK, MYB, MYC, MYD88, MYH9, MYSM1, NAF1, NBEAL2, NBN, NCF2, NCOR2, NF1, NHEJ1, NHP2, NLRP3, NOD2, NOP10, NOTCH1, NOTCH2, NPM1, NR3C1, NRAS, NT5C2, NT5C3A, NTRK3, NUP214, OS9, P2RY2, PALB2, PARN, PAX5, PBX1, PC, PCDHB1, PDGFRA, PDGFRB, PDHA1, PDHX, PFKL, PFKM, PGK1, PGM3, PHF6, PICALM, PIEZO1, PIGA, PIK3CD, PIK3R1, PKLR, PML, PMS2, PNP, POT1, PRDM1, PRF1, PRKACG, PRPF40B, PTCH2, PTEN, PTK2B, PTPN11, PTPN2, PTPRC, PTPRD, PUS1, RAB27A, RAC1, RAC2, RAD21, RAD50, RAD51, RAD51C, RAF1, RAG1, RAG2, RB1, RBBP6, RBM8A, RELN, RFWD3, RHAG, RHOA, RIT1, RMRP, RNF168, RPL10, RPL11, RPL15, RPL23, RPL26, RPL27, RPL31, RPL35A, RPL36, RPL5, RPS10, RPS14, RPS15, RPS17, RPS19, RPS24, RPS26, RPS27, RPS27A, RPS28, RPS29, RPS7, RTEL1, RUNX1, RUNX1T1, SAMD9L, SBDS, SBF2, SEC23B, SERPING1, SETBP1, SETD2, SF1, SF3A1, SF3B1, SH2B3, SH2D1A, SHOC2, SLC11A2, SLC19A2, SLC25A38, SLC2A1, SLC35C1, SLC37A4, SLC4A1, SLCO1B1, SLCO1B3, SLFN14, SLX4, SMARCD2, SMC1A, SMC3, SOS1, SPINK5, SPRED1, SPTA1, SPTB, SRC, SRCAP, SRP72, SRSF2, STAG1, STAG2, STAT3, STAT5B, STEAP3, STX11, STXBP2, SUZ12, SYNE1, TAL1, TAL2, TAZ, TBL1XR1, TBX1, TCF3, TCIRG1, TEC, TERC, TERF1, TERF2, TERF2IP, TERT, TET1, TET2, TET3, THBD, THPO, TINF2, TLX1, TLX3, TMPRSS6, TNFAIP3, TNFRSF13B, TNFRSF14, TNFRSF1A, TOX, TP53, TPI1, TPMT, TRAF3, TRNT1, TSLP, TSR2, TUBB1, TYK2, U2AF1, U2AF2, UBE2T, UGT1A1, UGT1A7, UNC13B, UNC13D, UNC5D, USB1, USH2A, USP9X, VHL, VPS13B, VPS45, VWF, WAS, WDR1, WIPF1, WRAP53, WT1, XBP1, XIAP, XK, XRCC2, YARS2, ZAP70, ZFHX4, ZNF197, ZRSR2, MRE11A, WHSC1, STON1, OBFC1

Table S2. Effects of Mdivi-1 on venetoclax-induced apoptosis in primary acute myeloid leukemia FLT3 cells and normal BMMCs

Patient No. #	Sex/Age	Chromosome	Mutation	Source	venetoclax (50nM, 48 h) + Mdivi-1 (20μM, 48 h)
			TP53		AnnexinV$^+$ PI$^+$, %
YH01	F/60	44~45,XX,-5,+6,t(8;12)(q24.1;p12),-10,der(16)t(16;17)(q24;q11.2),-17,+mar1,+mar2[cp15]	mut	BM	66.22
YH02	F/80	80~87,XXXX,+1~3mar,der(3),del(4),del(5),add(7)	mut	BM	54.49
YH03	M/62	46,XY,-8,der(9)del(9)(p21p21)del(9)(q22q31),der(10)t(10;?)(p11.2;?),del(17)(p11.2),+mar[11]/45,XY,-8,der(9)del(9)del(9),-10,der(10)t(10;?),i(17)(q10),+mar[10]/46,XY,+6,-8,der(9)del(9)del(9),-10,der(10)t(10;?),i(17)(q10),+mar[3]	mut	BM	58.58
YH04	M/57	46,XY,del(5)(q13q31),del(7)(q11.2),+11,der(12)t(1;12)(p13;q24.3),?psu dic(17;16)(p11.2;q12-13),?der(18)t(18;19)(q23;q13.1-13.3)[20]	mut	BM	71.23
YH05	F/74	44~49,XX,-4,der(5)t(5;?)(q12;?),+8,-11,del(11)(q13),der(12)t(12;?)(q13;?),-14,-18,-20,-22,+4~5mar[cp22]	mut	BM	66.81
Donor01	F/45	46, XY	Wildtype	BMMC	22.92
Donor02	M/18	46, XY	Wildtype	BMMC	17.18
Donor03	M/52	46, XY	Wildtype	BMMC	17.53
Donor04	F/37	46, XX	Wildtype	BMMC	21.33
Donor05	M/67	46, XY	Wildtype	BMMC	16.22

Note. mut, mutation; BM, bone marrow; BMMC, bone marrow mononuclear cells

Table S3. Brand, catalog no. and dilution of each antibody

Ab	Brand name	Catalog No.	Dilution
DRP1	Cell signaling	5391	1:1000
p-DRP1 (S616)	Cell signaling	3455	1:1000
p-DRP1 (S637)	abcam	ab193216	1:1000
TP53	Cell signaling	2527	1:1000
p21	Cell signaling	2947	1:1000
FIS1	Santa cruz	sc-376469	1:1000
OPA1	Cell signaling	80471	1:1000
MFF	abcam	ab129075	1:1000
MUL1	abcam	ab209263	1:1000
Cleaved caspase-3	Cell signaling	9661	1:1000
PARP	Cell signaling	9542	1:1000
Cytochrome C	BD	556533	1:1000
Bcl-2	Santa cruz	sc-509	1:1000
Mcl-1	Cell signaling	39224	1:1000
p-Mcl-1 (T163)	Cell signaling	14765	1:1000
Bcl-xL	Santa cruz	sc-7195	1:500
BIM	Cell signaling	2819	1:1000
NOXA	abcam	ab13654	1:1000
PUMA	Mybiosource	mbs435293	1:1000
Bax	Santa Cruz	sc-23959	1:1000
Bak	Santa Cruz	sc-517390	1:1000
CoxIV	abcam	ab14744	1:1000
b-actin	Santa Cruz	sc-69879	1:1000
