# Supplementary material for: DRP1 Inhibition Enhances Venetoclax-Induced Mitochondrial Apoptosis in TP53-Mutated Acute Myeloid Leukemia Cells through BAX/BAK Activation
Source: Cancers (Basel). 2023 Jan 25;15(3):745. doi: 10.3390/cancers15030745 (PMC9913445; doi:10.3390/cancers15030745)
Supplement: Supplementary file 1 [file cancers-15-00745-s001.zip › cancers-2174698-supplementary/Uncropped version for WB ver2.pptx]

## Slide 1
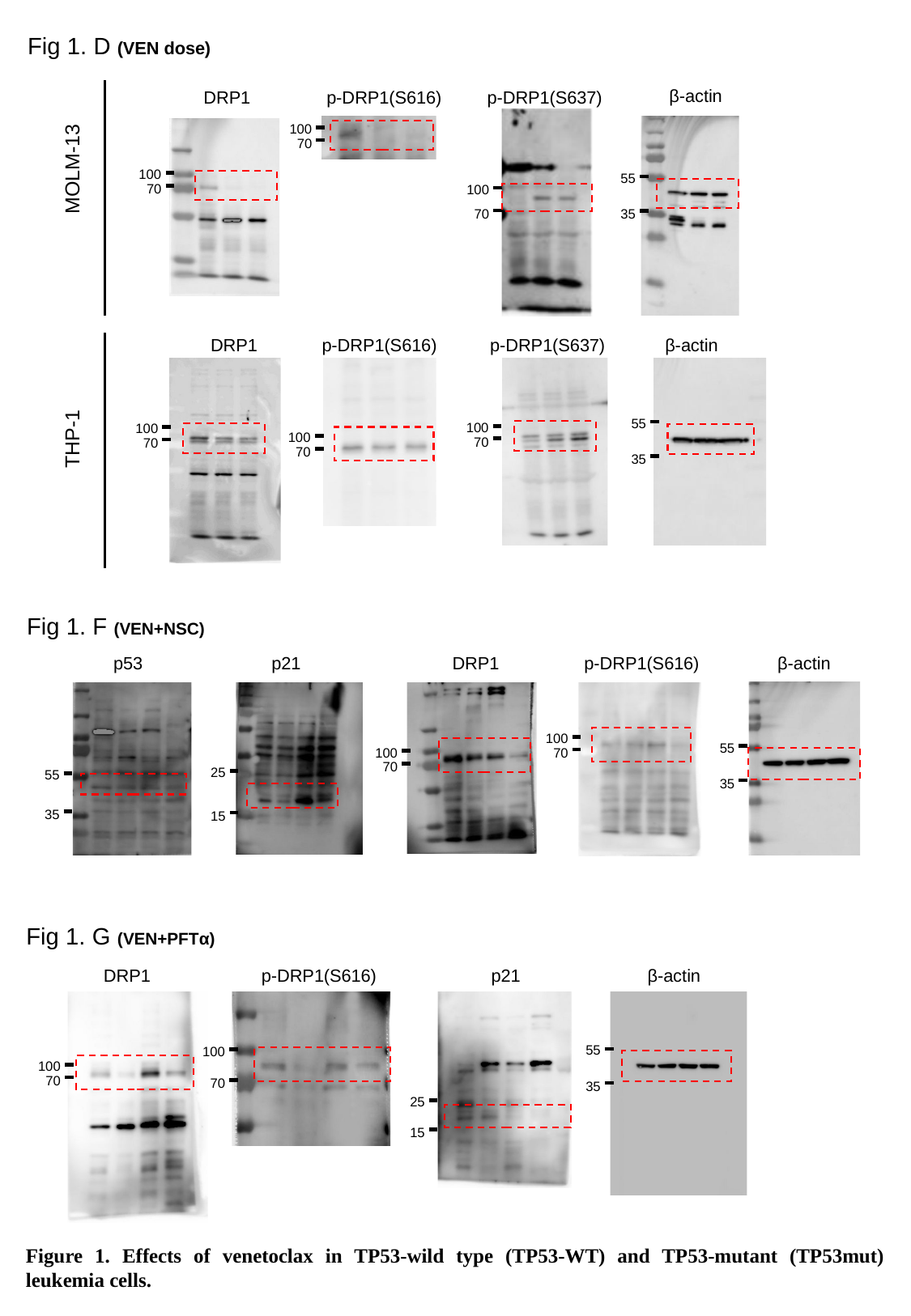

Fig 1. D (VEN dose)
β-actin
DRP1
p-DRP1(S616)
p-DRP1(S637)
100
70
MOLM-13
100
55
70
100
70
35
DRP1
p-DRP1(S616)
p-DRP1(S637)
β-actin
55
100
100
THP-1
100
70
70
70
35
Fig 1. F (VEN+NSC)
p53
p21
DRP1
p-DRP1(S616)
β-actin
100
55
100
70
70
25
55
35
35
15
Fig 1. G (VEN+PFTα)
DRP1
p-DRP1(S616)
p21
β-actin
55
100
100
70
70
35
25
15
Figure 1. Effects of venetoclax in TP53-wild type (TP53-WT) and TP53-mutant (TP53mut) leukemia cells.

## Slide 2
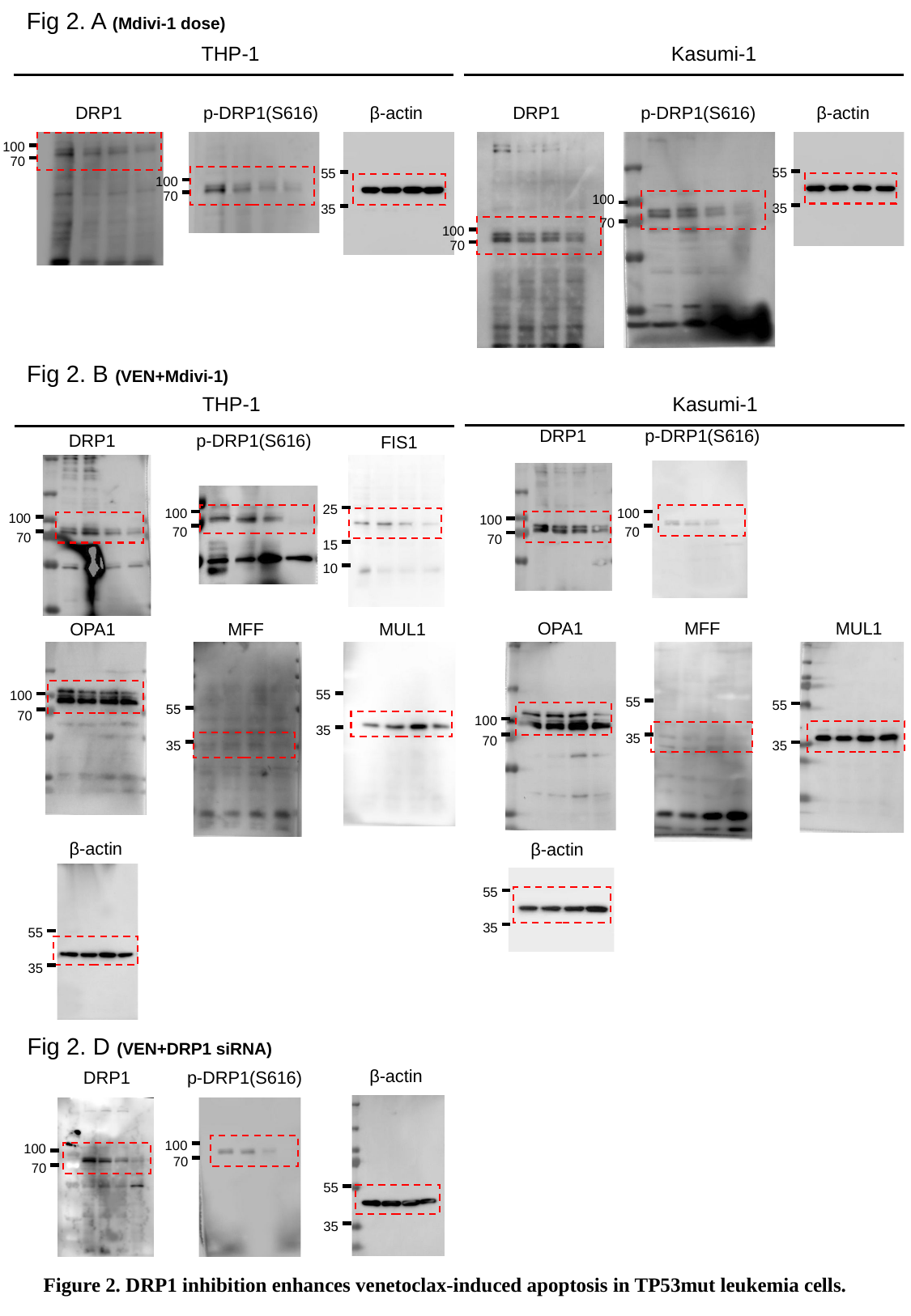

Fig 2. A (Mdivi-1 dose)
THP-1
Kasumi-1
DRP1
p-DRP1(S616)
β-actin
DRP1
p-DRP1(S616)
β-actin
100
70
55
55
100
70
100
35
35
70
100
70
Fig 2. B (VEN+Mdivi-1)
THP-1
Kasumi-1
DRP1
p-DRP1(S616)
DRP1
p-DRP1(S616)
FIS1
25
100
100
100
100
70
70
70
70
15
10
OPA1
MFF
MUL1
OPA1
MFF
MUL1
55
100
55
55
55
70
100
35
35
70
35
35
β-actin
β-actin
55
35
55
35
Fig 2. D (VEN+DRP1 siRNA)
β-actin
DRP1
p-DRP1(S616)
100
100
70
70
55
35
Figure 2. DRP1 inhibition enhances venetoclax-induced apoptosis in TP53mut leukemia cells.

## Slide 3
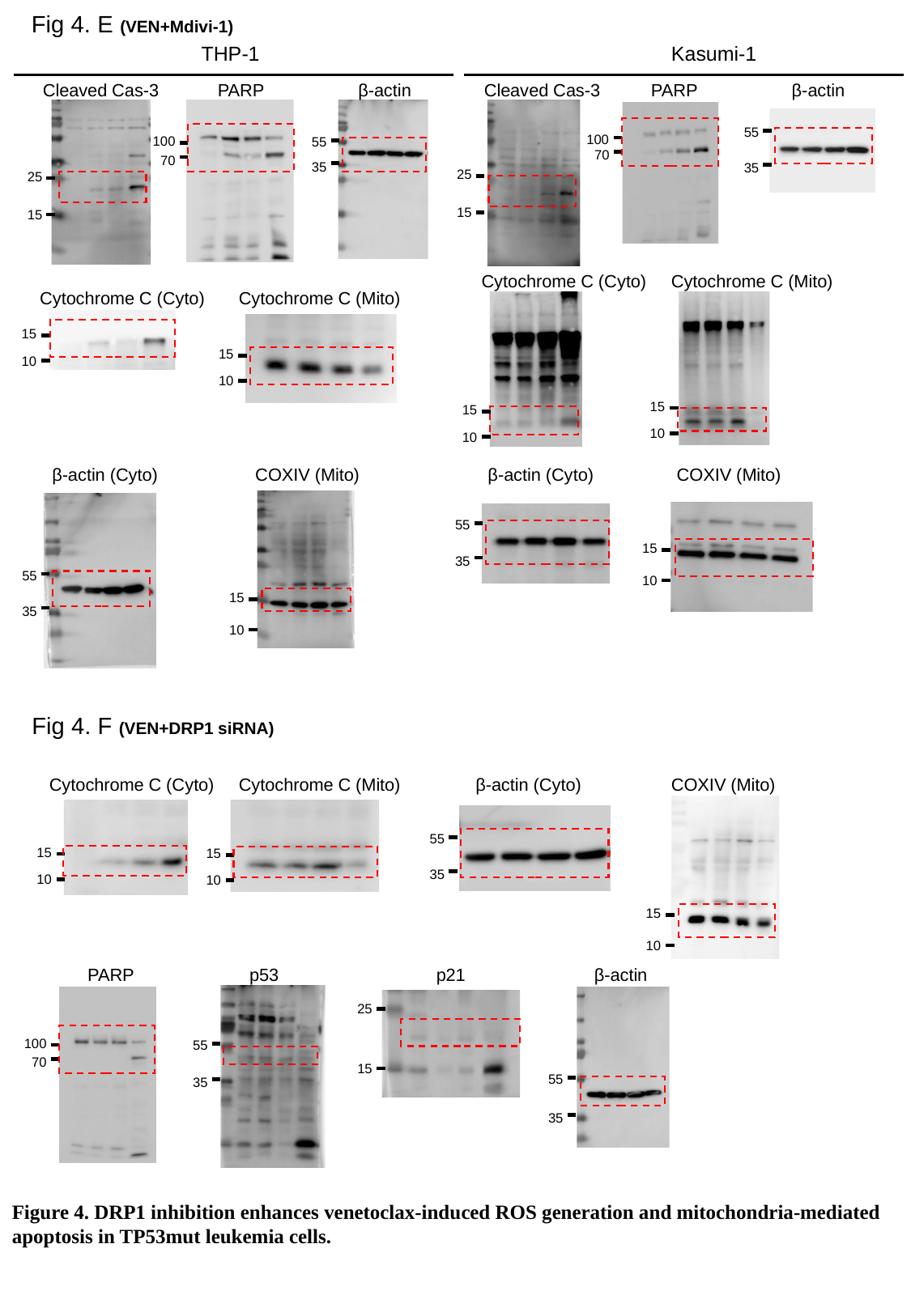

Fig 4. E (VEN+Mdivi-1)
THP-1
Kasumi-1
Cleaved Cas-3
PARP
β-actin
Cleaved Cas-3
PARP
β-actin
55
100
100
55
70
70
35
35
25
25
15
15
Cytochrome C (Cyto)
Cytochrome C (Mito)
Cytochrome C (Mito)
Cytochrome C (Cyto)
15
15
10
10
15
15
10
10
β-actin (Cyto)
COXIV (Mito)
β-actin (Cyto)
COXIV (Mito)
55
15
35
55
10
15
35
10
Fig 4. F (VEN+DRP1 siRNA)
Cytochrome C (Cyto)
Cytochrome C (Mito)
β-actin (Cyto)
COXIV (Mito)
55
15
15
35
10
10
15
10
PARP
p53
p21
β-actin
25
100
55
70
15
55
35
35
Figure 4. DRP1 inhibition enhances venetoclax-induced ROS generation and mitochondria-mediated apoptosis in TP53mut leukemia cells.

## Slide 4
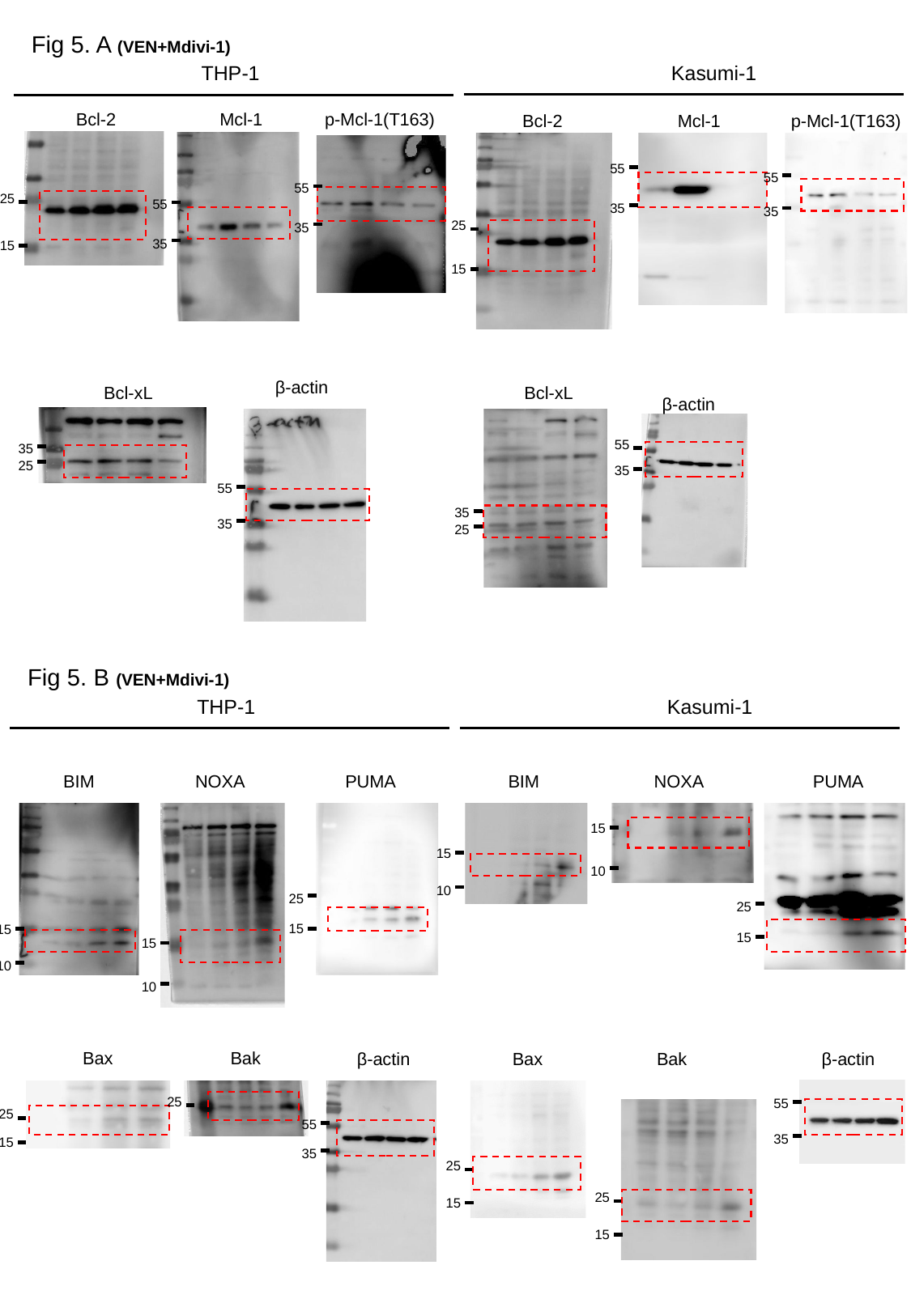

Fig 5. A (VEN+Mdivi-1)
THP-1
Kasumi-1
Bcl-2
Mcl-1
p-Mcl-1(T163)
Bcl-2
Mcl-1
p-Mcl-1(T163)
55
55
55
25
55
35
35
25
35
35
15
15
β-actin
Bcl-xL
Bcl-xL
β-actin
55
35
25
35
55
35
35
25
Fig 5. B (VEN+Mdivi-1)
THP-1
Kasumi-1
BIM
NOXA
PUMA
BIM
NOXA
PUMA
15
15
10
10
25
25
15
15
15
15
10
10
Bax
Bak
β-actin
Bax
Bak
β-actin
25
55
25
55
35
15
35
25
25
15
15

## Slide 5
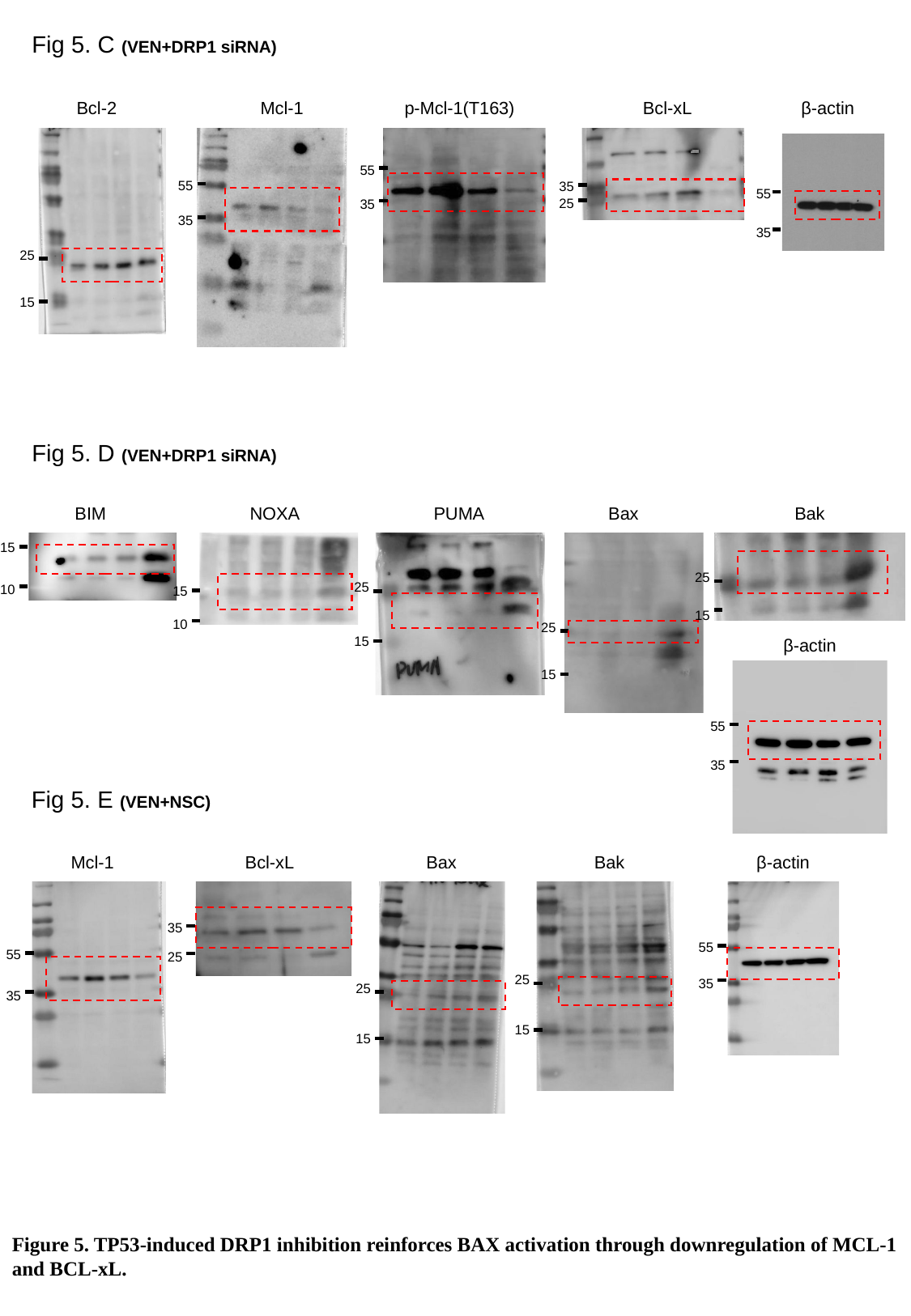

Fig 5. C (VEN+DRP1 siRNA)
Bcl-2
Mcl-1
p-Mcl-1(T163)
Bcl-xL
β-actin
55
55
35
55
25
35
35
35
25
15
Fig 5. D (VEN+DRP1 siRNA)
BIM
NOXA
PUMA
Bax
Bak
15
25
25
10
15
15
10
25
15
β-actin
15
55
35
Fig 5. E (VEN+NSC)
Mcl-1
Bcl-xL
Bax
Bak
β-actin
35
55
55
25
25
35
25
35
15
15
Figure 5. TP53-induced DRP1 inhibition reinforces BAX activation through downregulation of MCL-1 and BCL-xL.

## Slide 6
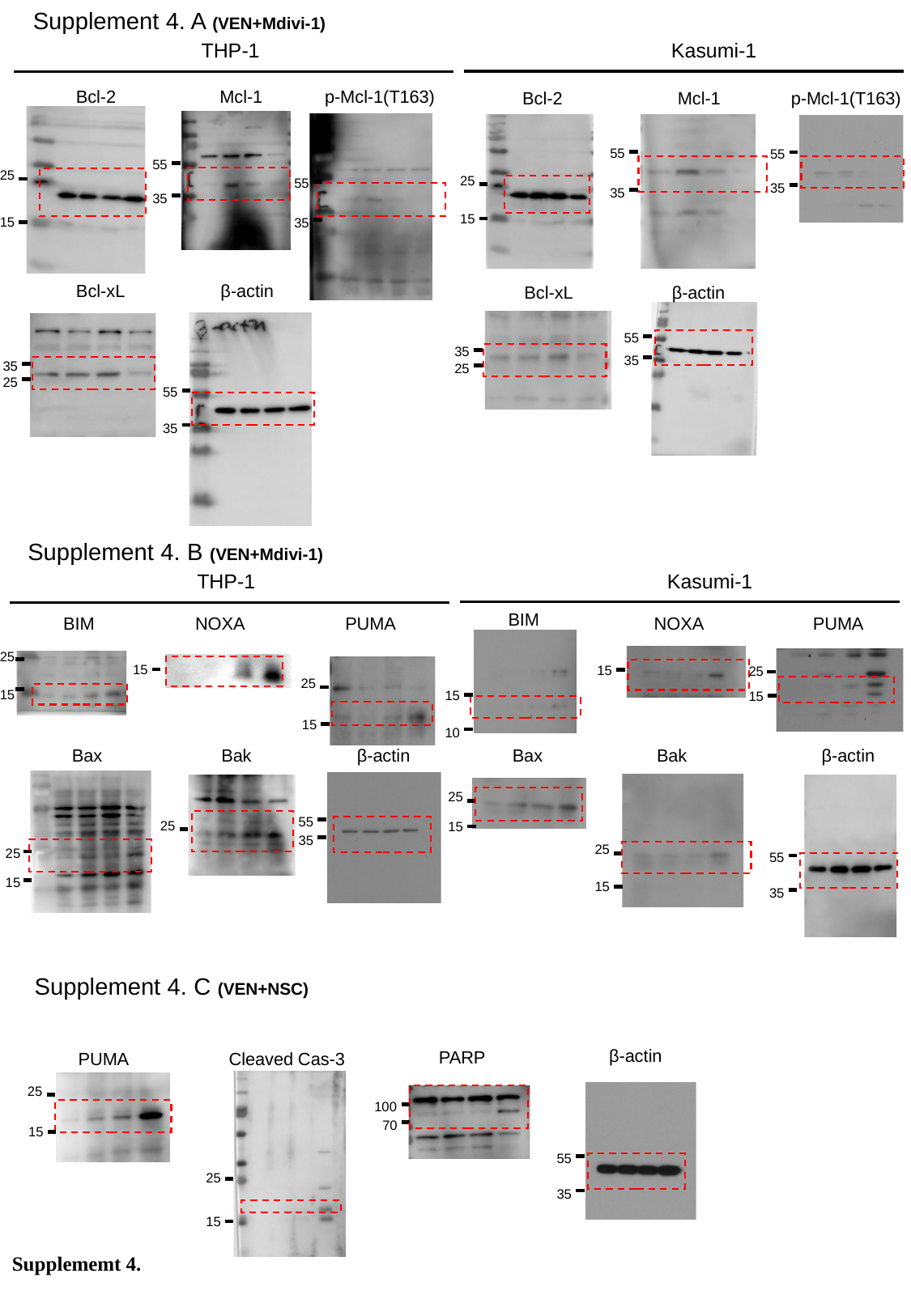

Supplement 4. A (VEN+Mdivi-1)
THP-1
Kasumi-1
Bcl-2
Mcl-1
p-Mcl-1(T163)
Bcl-2
Mcl-1
p-Mcl-1(T163)
55
55
55
25
25
55
35
35
35
15
15
35
Bcl-xL
β-actin
Bcl-xL
β-actin
55
35
35
35
25
25
55
35
Supplement 4. B (VEN+Mdivi-1)
THP-1
Kasumi-1
BIM
BIM
NOXA
PUMA
NOXA
PUMA
25
15
15
25
25
15
15
15
15
10
Bax
Bak
β-actin
Bax
Bak
β-actin
25
55
25
15
35
25
25
55
15
15
35
Supplement 4. C (VEN+NSC)
β-actin
PARP
PUMA
Cleaved Cas-3
25
100
70
15
55
25
35
15
Supplememt 4.
